# Exploring Emotional Stimuli Detection in Artworks: A Benchmark Dataset and Baselines Evaluation †

**DOI:** 10.3390/jimaging10060136

**Published:** 2024-06-04

**Authors:** Tianwei Chen, Noa Garcia, Liangzhi Li, Yuta Nakashima

**Affiliations:** 1 Intelligence and Sensing Lab, Osaka University, Suita, Osaka 565-0871, Japan; noagarcia@ids.osaka-u.ac.jp (N.G.); n-yuta@ids.osaka-u.ac.jp (Y.N.); 2 Computer Science Department, Qufu Normal University, Qufu 273165, China; liliangzhi@ieee.org

**Keywords:** emotional stimuli detection, artwork analysis

## Abstract

We introduce an emotional stimuli detection task that targets extracting emotional regions that evoke people’s emotions (i.e., emotional stimuli) in artworks. This task offers new challenges to the community because of the diversity of artwork styles and the subjectivity of emotions, which can be a suitable testbed for benchmarking the capability of the current neural networks to deal with human emotion. For this task, we construct a dataset called APOLO for quantifying emotional stimuli detection performance in artworks by crowd-sourcing pixel-level annotation of emotional stimuli. APOLO contains 6781 emotional stimuli in 4718 artworks for validation and testing. We also evaluate eight baseline methods, including a dedicated one, to show the difficulties of the task and the limitations of the current techniques through qualitative and quantitative experiments.

## 1. Introduction

Analyzing artworks in machine learning is a challenging task. Compared to photographs, artworks do not only depict real-world concepts, such as humans, animals, and natural scenes, but also represent humane contents, such as feelings, attitudes, and faiths. The richness in the representations and the diversity of styles make artworks the ideal testbed to study new challenges related to human emotion understanding in machine learning.

In recent years, many efforts have been undertaken in the field of artwork analysis [1,2,3,4,5,6,7,8,9,10], aiming to improve models’ understanding of artworks and further extend models’ capacity to support digital humanities, with tasks such as attribute identification [1,5,6,7,8], object detection [3,4], or artwork understanding through language [2,9,10]. Thanks to these studies, recent models have developed a reliable capacity to understand *objective* contents (e.g., objects, attributes, and descriptions) from the artworks. However, only a few studies [11,12] are focusing on a more *subjective* and personal analysis, such as the relationship between artwork and emotions.

ArtEmis [11] and its extension ArtEmis V2.0 [12] are two datasets collected for studying the relationship between artworks and emotions. The main focus is on the generation of emotional captions that can accurately capture the emotional influence of an artwork. However, a more in-depth analysis to uncover why and how emotions are evoked from the artworks is still not explored. In other words, ArtEmis and ArtEmis 2 show that models can generate emotional captions, but it is still unknown how the emotions are evoked from those artworks.

**Figure 1:**
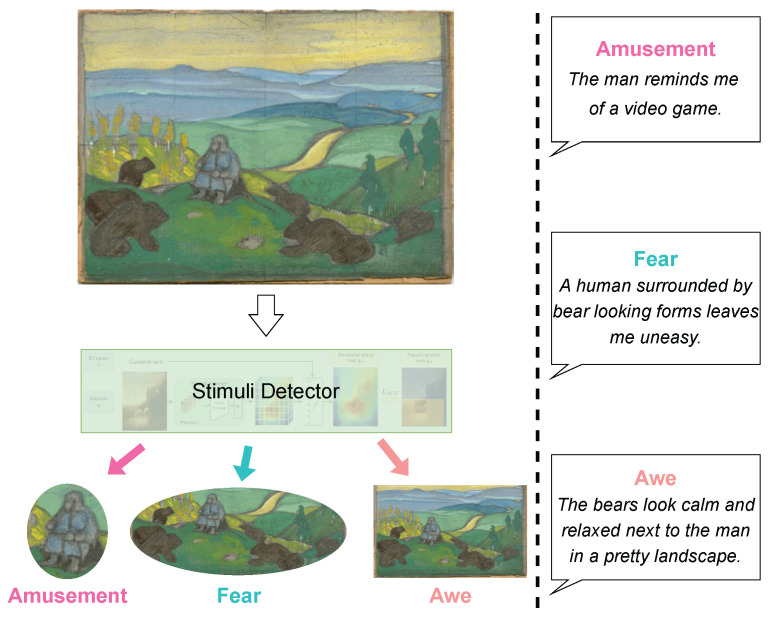
A model detects emotional regions that evoke people’s emotions (namely, emotional stimuli) from the given artwork. The utterances on the right side may be used as hints to spot emotional stimuli.

Artworks can easily elicit people’s emotions, yet this elicitation process is complex and underexplored [13,14,15,16]. The appraisal theory toward artworks and emotions [13] says the emotion-evoking process is related to the viewer’s analysis process; the emotions are evoked during the viewer’s analysis process through the whole artwork. Thus, different analyses may lead to different emotions. For example, given the artwork in Figure 1, people may feel different emotions when the analysis concentrates on different visual concepts in the context of the artwork. If a viewer focuses on the distorted style of the person, a feeling of amusement may be evoked, while the bear-like brown figures may be linked with fear. Learning such processes could make models acquire knowledge about how human emotions are evoked and may improve models’ capacity to utilize emotional stimuli. Such merits could be helpful for tasks related to emotions (e.g., visual emotion recognition [17,18,19,20,21]) and tasks potentially involving emotion analysis (e.g., image generation [22,23]).

According to these observations, we propose a new task for emotional stimuli detection in artworks, in which a model is required to detect emotional stimuli from a given artwork, as shown in Figure 1. The task, which explores a machine’s capacity to understand emotions and artworks, has two major challenges: First, different from photorealistic images, *artworks are painted with a certain style*. For example, in Western art, Realism is one of the styles that may look more like a real photo, while Impressionism typically shows prominent brush strokes. Different styles lead to very different appearances of the same object. This style variation makes it harder to learn visual content (e.g., objects) from artworks than photos [1,4,24]. Second, *emotions are subjective*. Different people may have different emotions evoked by the same artwork [11,12]. This subjectivity makes the task unique, as an artwork can have multiple emotional stimuli for different emotions.

For this task, we construct a benchmark dataset, coined APOLO (**A**rtwork **P**rovoked em**O**tion Eva**L**uati**O**n), to evaluate models in both qualitative and quantitative ways. We build APOLO on top of the ArtEmis dataset [11], which, for each artwork, includes emotion labels annotated by multiple annotators, and utterances (sentences) that explain why emotions are provoked. To further understand the stimuli that provoke emotions, APOLO includes pixel-level emotional stimuli annotations on the images of the test set. As a result, we collect 6781 emotional stimuli for 4718 artworks and eight emotions. Our exhaustive control quality checks ensure the samples are balanced and reliable. To the best of our knowledge, this is the first dataset that offers pixel-level annotations of emotional stimuli in artworks.

Additionally, we explore multiple models for emotional stimuli detection, borrowed from related tasks, including object detection, referring expression, and saliency map detection. We also introduce a dedicated weakly supervised model as a baseline, which predicts emotional stimuli regions for each emotion without using region-level annotations for training. Our comprehensive experiments on APOLO show that the evaluated models can detect emotional stimuli even when not trained with region annotations. However, the emotional stimuli detection task is still challenging and has plenty of room for improvement. In addition, we explore how a text-to-image generative model, Stable Diffusion [22], handles emotions in the input prompts, observing that it fails to connect the emotional words in the input with the emotional stimuli in the generated images. We hope our work will help overcome this limitation in the future. Our data and code are available at https://github.com/Tianwei3989/apolo.

## 2. Related Work

*Visual emotion analysis* Given an input image, visual emotion analysis aims to recognize emotions, analyze the emotional stimuli, and apply the recognized emotions to real-world applications (e.g., psychological health [25,26] and opinion mining [27,28]) to improve the ability of emotional intelligence [29]. Most of the recent studies [17,18,19,20,21] use emotional stimuli to improve emotion recognition, but only a few efforts have been made to analyze how well the models detect such stimuli. To the best of our knowledge, only two datasets, the EmotionROI [30] and EMOd [31], provide pixel-level annotations for evaluating emotional stimuli detection. However, they are both relatively small, offering 1980 and 1019 labeled images, respectively, consisting of social media images from the Internet.

Data scarcity is one of the main challenges in emotional stimuli detection. To overcome this problem, we propose two solutions: (1) transferring models from related tasks and (2) designing a weakly supervised learning model that does not require costly pixel-level annotations for training. For evaluation, we collect a dataset with emotional stimuli annotations.

*Artwork analysis* Much effort has been dedicated to solving art-related problems with machine learning techniques, including style identification [1,32], object detection [1,3,4], instance-level recognition [33], or artwork description [2,9,34]. Concerning emotion analysis, some datasets [1,11,12,35], including ArtEmis, contain labels with the emotion (e.g., *amusement* and *fear*) that each artwork evokes. Nevertheless, the same artwork can evoke multiple emotions according to different regions of the image, a fact that has been unexplored in current datasets. APOLO introduces a new challenge by investigating the connection between artworks and emotion at the pixel level.

## 3. Emotional Stimuli Detection

Our task aims to explore how a model can find the cues of the emotion elicitation process from the artwork, i.e., the emotional stimuli. In general, we explore two separate scenarios: (1) emotional stimuli detection *without reference* (i.e., utterances) and (2) emotional stimuli detection *with reference*. Ideally, a model should find emotional stimuli without reference, like humans can. However, such models are rare since only a few studies are aimed at emotional stimuli detection. We thus also explore whether recent multimodal models can detect emotional stimuli by using the references.

Formally, let *a*, e∈E, and *u* denote an artwork, its emotion label, and the utterance, which can be a set of sentences, in ArtEmis, where E is the set of the emotions. $D_{t}$ denotes the training set of ArtEmis [11], where $D_{t}$ contains triples (a,e,u). $D_{v}$ and $D_{e}$ denote the validation and test sets of APOLO, where both contain triples (a,e,u). As we presume that an artwork can evoke potentially *any* emotion depending on where the viewer focuses their attention, the emotional stimuli detection task can be formulated as a segmentation task given artwork *a* and emotion e∈E, in which a model $f_{e}$ predicts segment *s* that evoke emotion *e* as

(1) $$\begin{array}{c} \hat{s}=f_{e}\left(a\right), \end{array}$$

where $\hat{s}$ is the predicted segment.

This task can be extremely challenging as no cue is provided for specifying the regions that are involved given emotion *e*. We thus formulate a variant with reference by *u*, in which *u* is given to a model as an auxiliary cue for emotional stimuli detection, i.e.,

(2) $$\begin{array}{c} \hat{s}=f_{e}\left(a,u\right). \end{array}$$

In both scenarios (emotional stimuli detection with and without reference), we can use $D_{t}$ for training a model, but ground-truth segments are not available. $D_{v}$ and $D_{e}$ are solely used for validation and testing.

## 4. APOLO Dataset Curation

APOLO is a benchmark dataset for evaluating, both quantitatively and qualitatively, emotional stimuli detection in artworks. We utilize the test samples in ArtEmis [11], with 39,850 explanatory utterances and emotional responses related to 8003 artworks from WikiArt (https://www.wikiart.org/ (accessed on 30 May 2024)).

**Figure 2:**
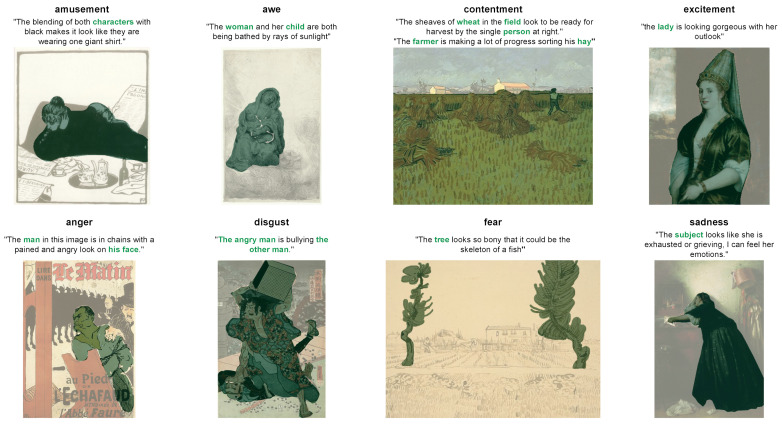
Some samples in our dataset. The words and regions in green are the chosen noun phrases and the annotated emotional stimuli, respectively. If one artwork-emotion pair contains multiple utterances, the corresponding regions are then combined. We annotate regions for eight emotions from ArtEmis, except “something else”.

ArtEmis is annotated with nine emotions: *amusement*, *anger*, *awe*, *contentment*, *disgust*, *excitement*, *fear*, *sadness*, and *something else*. As shown in Figure 2, the utterances are explanations of why a certain emotion is evoked by the artwork, which is usually related to its emotional stimuli.

We observe that the utterances potentially align with the viewers’ analysis processes and are related to a certain emotion that is specified by the emotion label and evoked by the artwork. Furthermore, the utterances tend to describe certain regions, which leads to a certain emotion, in the artwork. These features may help models to learn how humans perceive emotion from or associate emotion with such regions.

**Figure 3:**
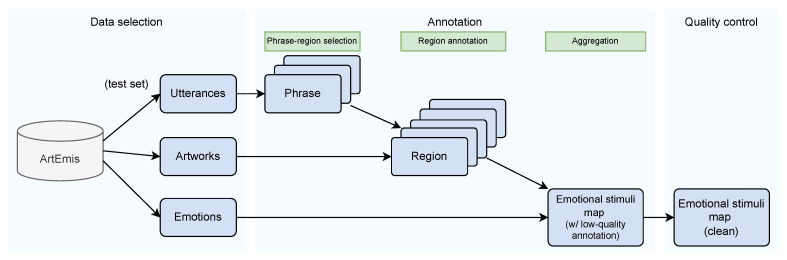
The workflow of APOLO dataset curation. In general, APOLO is extended from the ArtEmis dataset. We collect the annotation from ArtEmis’s test set and further annotate the pixel-level emotional stimuli map from the artworks.

Toward this end, we construct a pixel-level emotional stimuli dataset, APOLO, by asking 91 annotators in Amazon Mechanical Turk (https://www.mturk.com/) (accessed on 30 May 2024) to identify the visual concepts that involve the utterances and to annotate the visual concepts at the pixel level, as shown in Figure 2 and Figure 3. We show its details in Table 1.

We only collect validation and test sets by randomly sampling ArtEmis’s test set, considering (1) the evaluation could be applied to recent large models (e.g., CLIP [36]) that are hard to train and (2) the cost of pixel-level annotation. During the APOLO dataset curation, we always use random selection as our selection principle.

### 4.1. Data Selection

To curate our annotations from ArtEmis, we annotate paintings from the first eight emotions from ArtEmis’s nine emotions, i.e., the emotions of *amusement*, *anger*, *awe*, *contentment*, *disgust*, *excitement*, *fear*, and *sadness*. We filter out samples with the *something else* label, as we found from the associated utterance that their interpretation of the emotion is not trivial and annotators may not capture clear ideas from them. For each of the other eight emotion labels, we randomly choose about 1200 artwork–utterance pairs from the ArtEmis test set, except for the emotion *anger*, which only contains 672 artwork–utterance pairs. Overall, we select 9599 samples.

### 4.2. Annotation Process

**Figure 4:**
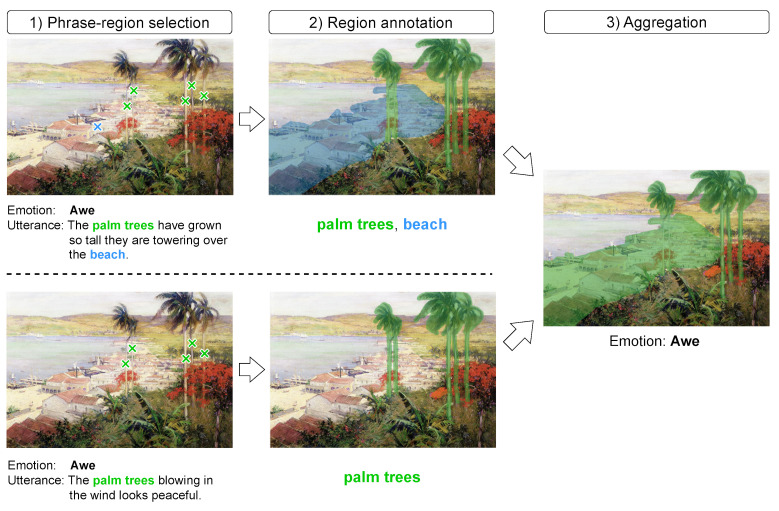
In our annotation process, workers should annotate the following three steps: (1) phrase-region selection, (2) region annotation, and (3) aggregation. The green and blue ×’s in the first step are the location of the regions. We randomly check the submissions at every step to ensure the annotation quality.

As the aim is to annotate emotional stimuli, which are regions that can evoke a certain emotion, we design an annotation process focused on identifying the regions that correspond to specific phrases in the utterances, as these phrases are strongly tied to the emotions. The general annotation process is shown in Figure 4, and it consists of three steps: (1) phrase-region selection, (2) region annotation, and (3) aggregation.

**Figure 5:**
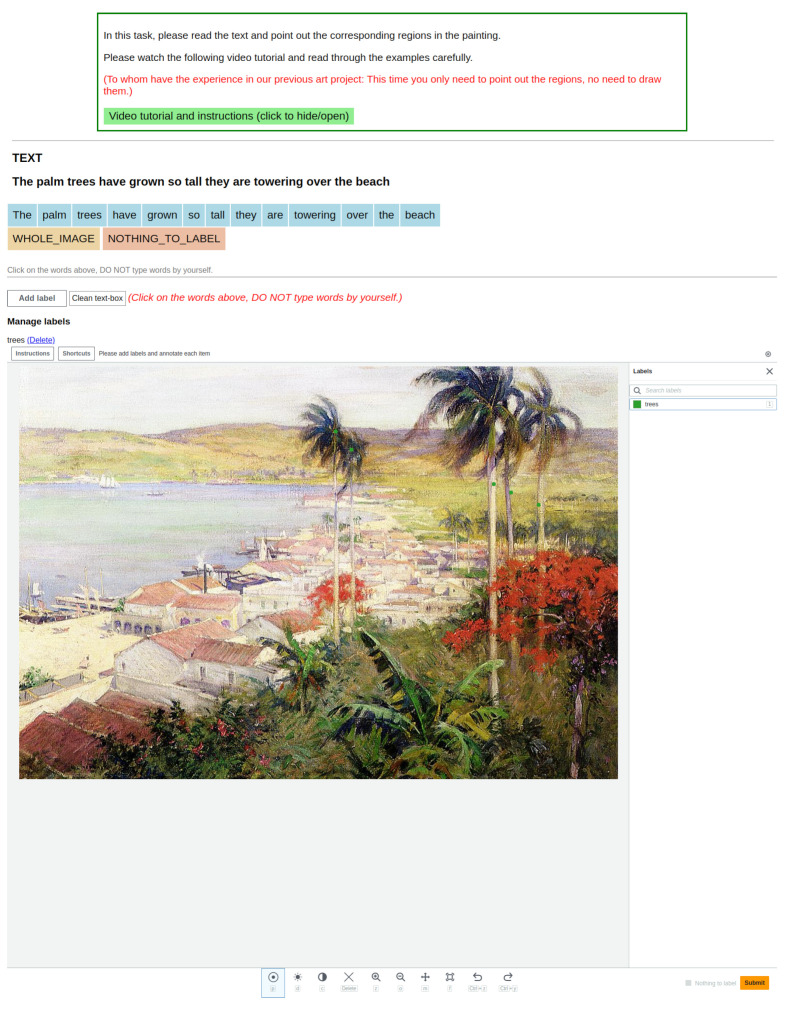
Annotation interface of phrase-region selection. On this interface, an annotator should first read the utterance and artwork and then point out the location of the region. The blue, yellow, and orange blocks in the “TEXT” section are the buttons for annotators to select.

*Phrase-region selection* The annotation interface is shown in Figure 5. In the first step, we aim to gather the cues of the emotion elicitation process from the utterances, i.e., to collect phrases in the utterances that correspond to the emotional stimuli and their corresponding location on the artwork.

To identify such phrases, we show annotators a single utterance *u* together with an artwork *a*. Note that by design, ArtEmis utterances *u* explicitly describe the emotion generated by the artwork *a*. Then, we ask them to find all the noun phrases in *u* that explicitly mention visual concepts in *a*. We denote the set of identified phrases in *u* by $W_{u}$, where $w\in W_{u}$ is a phrase (e.g., the “trees”). Specifically, we provide annotators two additional options, the *whole artwork* and the *nothing to label* (the yellow and orange buttons as shown in Figure 5), since some utterances may only talk about the whole image or nothing related to the artwork. If there is at least one phrase in the utterances that corresponds to the emotional stimuli, the annotators are then asked to locate the region in the artwork by spotting at least one point that lies in the region of the visual concepts by clicking on the artwork in our annotation interface. The set of points for phrase *w* is denoted by $P_{w}$, where $p\in P_{w}$ is in $R^{2}$. To ensure that all phrases in the utterance and visual concepts are found, and also to reduce the subjectivity of annotation, we ask for three annotators per triplet (a,e,u) and aggregate annotations by removing duplicates to form $W_{u}$ and $P_{w}$ for all $w\in W_{u}$. By this step, we collect two types of annotations: (1) the noun phrases that are related to both the artworks and the evoked emotions (the colored phrases in Figure 4) and (2) the locations of the region that the noun phrases (the green and blue ×’s in Figure 4).

*Region annotation* In this step, we aim to identify the regions related to the emotion elicitation process, i.e., to draw pixel-level annotations according to the utterances. We collect these annotations based on the locations in the previous step. We show *a*, *u*, *w*, and $P_{w}$ to an annotator and ask them to draw on top of *a* all pixels that fall into the visual concepts identified by $w\in W_{u}$ and $P_{w}$, obtaining a segment $s_{w}$, which is a set of pixels. By this step, we collect pixel-level annotations of the regions for each of the noun phrase (the colored regions in Figure 4).

*Aggregation* Next, we aggregate phrase-wise region annotations $s_{w}$ belonging to the same *a* and emotion *e*. For all *w* that is associated with *a* and *e*, i.e., $w\in W=\{w\in W_{u^{\prime }}|\left(a^{\prime },e^{\prime },u^{\prime }\right)\in D,a=a^{\prime },e=e^{\prime }\}$, we obtain the aggregated emotional stimulus *s* by

(3) $$\begin{array}{c} s=\underset{w\in W}{⋃}s_{w}. \end{array}$$

By this step, we finally collect the region annotations for each of the emotion. Some examples of *a*, *u*, and *s* are shown in Figure 2. As a result, we obtain 7512 emotional stimuli in 5160 artworks. The data structure is shown in Table 2.

### 4.3. Quality Control

We apply quality controls both during and after the annotation process. During the annotation process, we randomly check 10% of the annotations in every round of submission and reject the *dishonest* ones (e.g., phrase w∈W is wrong, region $s_{w}$ is wrong, etc.). After the annotation process, we manually check all the annotations with special attention to the following three cases: (1) when the *whole artwork* is annotated as a region, (2) when the annotation is *low-quality* (e.g., only draw the contour) or wrong (e.g., draw wrong regions), and (3) when no region (denoted *void*) is annotated in the artwork. We found 1211 instances of *whole artwork*, 33 of *low-quality*, and 87 of *void* annotations. We remove all of them from our dataset. Finally, to ensure that the dataset is balanced and the *whole artwork* annotations are not over-represented, we randomly remove 600 *whole artwork* annotations to form our APOLO dataset.

### 4.4. Evaluation Dataset Analysis

**Figure 6:**
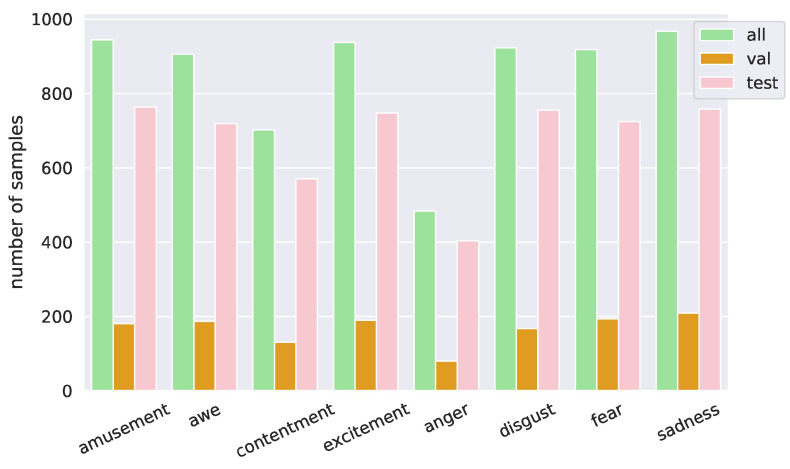
Emotion distribution of APOLO.

APOLO consists of 6781 emotional stimuli for 4718 artworks. We split it into validation and test sets with approximately 20% and 80% of the samples, respectively. The artworks in the validation and the test sets are disjointed. Figure 6 shows the distribution of emotion label *e* in APOLO. We remark that seven out of eight emotions have more than 500 samples, while the number of *anger* samples is smaller due to the fewer samples in the original ArtEmis dataset. The distributions of the validation and test sets are similar to that of the entire dataset.

**Figure 7:**
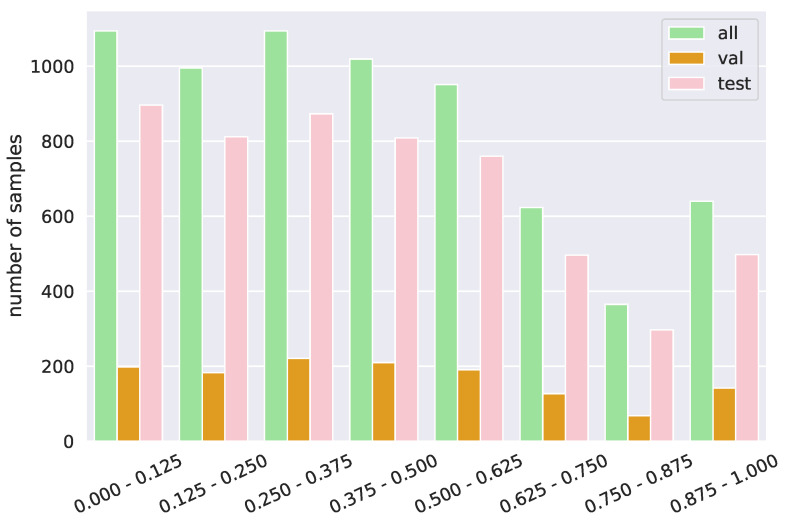
Stimuli occupation distribution of APOLO. The *x*-axis is the ratio of annotated pixels to the whole image, i.e., the occupation of the stimuli.

We also calculate the distribution of the ratio of pixels in *s* over *a*, i.e., |s|/|a|, where |·| gives the number of pixels in the region *s* or artwork *a*. Figure 7 shows the distribution. Many regions (46.94%) are small (|s|/|a|≤0.375), and less regions (24.01%) are large (|s|/|a|>0.625). From this, our evaluation dataset tends to have regions that focus on local concepts. The distributions of |s|/|a| for the validation and test sets are also similar to the entire dataset. Similar to ArtEmis, one artwork may contain a varied number (from one to eight) of emotions, and one artwork–emotion pair may contain a varied number of utterances.

## 5. Baselines

To better comprehend the challenges of the emotional stimuli detection task, we propose and evaluate several baselines.

### 5.1. Baselines with Reference

**Figure 8:**
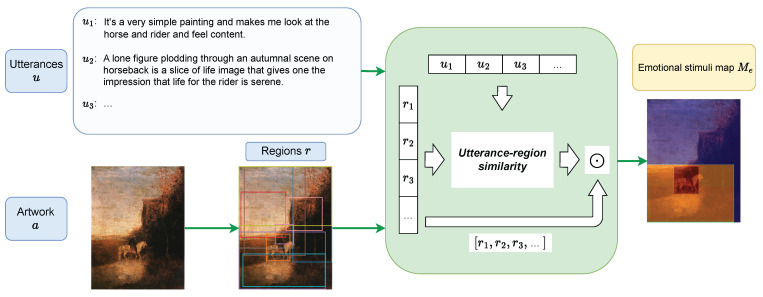
The overview of emotional stimuli map generation for baselines with reference.

In the with-reference variant, utterance *u* provides abundant information about what a model should look for, which reduces the task close to visual grounding, like refCOCO [37] and refCOCOg [38]. Our strategy is first to find regions relevant to *u* with utterance-region similarities and to weight the regions with the similarity to obtain a *emotional stimuli map* with pixel-level scores for *e*. This process is shown in Figure 8. Prediction $\hat{s}$ can be generated by thresholding the map.

We employ **VilBERT** [39] and **12-in-1** [40] as baselines, where 12-in-1 may have a variety of knowledge as it is trained over 12 vision-and-language tasks, while VilBERT is pre-trained on a large-scale dataset GCC [41]. To adapt to our task, VilBERT and 12-in-1 models are fine-tuned with refCOCO. These models give the probability of each region proposal given *u*, which can be interpreted as an utterance-region similarity score. **CLIP + VinVL** is a combination of CLIP [36] and VinVL [42]. CLIP [36] is renowned for its zero-shot capacity to solve vision-and-language tasks. We can first use VinVL to find region proposals and use CLIP to compute the utterance-region similarity with *u*.

*Emotional stimuli map generation* Let *R* denote the set of regions obtained from any of the above methods, and sim(r,u) be the utterance-region similarity between r∈R and *u*. We aggregate all regions in *R* to generate an *emotional stimuli map* $M_{u}$ for *u* by

(4) $$\begin{array}{c} M_{u}=\sum_{r\in R}sim\left(r,u\right)m_{r}, \end{array}$$

where $m_{r}$ is a map that represents *r* by giving 1 if a pixel in $m_{r}$ is in *r* and 0 otherwise. As an artwork *a* can be associated with multiple utterances for the same emotion, we aggregate all of them to obtain emotional stimuli map $M_{e}$ for *e* as

(5) $$\begin{array}{c} M_{e}=\sum_{u\in U_{ae}}M_{u}, \end{array}$$

where $U_{ae}=\left\{u^{\prime }|\left(u^{\prime },a^{\prime },e^{\prime }\right)\in D,a^{\prime }=a,e^{\prime }=e\right\}$. Thresholding is applied to $M_{e}$.

### 5.2. Baselines without Reference

#### 5.2.1. Object Detection

One naïve idea for the without-reference task is to spot salient regions in some senses and give the regions as emotional stimuli regardless of given emotion *e*. Object detection can give such regions [43]. We adopt the region proposal networks in **FasterRCNN** [44] and **VinVL** [42]. VinVL’s region proposal network may offer better performance as it can additionally detect some attributes (e.g., *blue* and *calm*) that may exhibit stronger ties with some emotions. We aggregate proposals with top-*K* confidence to form $\hat{s}$ for any e∈E (i.e., $f_{e}\left(a\right)=f_{e^{\prime }}\left(a\right)$ even for $e\neq e^{\prime }$). To obtain segment prediction $\hat{s}$, we follow the same procedure as emotional stimuli map generation in the previous section, but we use 1/|r| in place of sim(r,u) as this task does not allow us to use *u*, so we cannot compute sim(r,u).

#### 5.2.2. CASNet and CASNet II

**CASNet** [31] is a learning-based model for saliency detection, which generates a saliency map for a given image. The model is trained on a dataset called EMOd, which contains images that evoke some emotions and human fixations. With this dataset, CASNet learns to find regions that draw human attention. The work [31] showed, based on their analysis over EMOd, that humans tend to focus more on *emotional* objects than on *neutral* objects, where *emotional* and *neutral* objects are annotated by annotators. Therefore, CASNet also tends to focus on *emotional* objects. For our task, we apply thresholding to the saliency map to obtain $\hat{s}$. Again, prediction $\hat{s}$ is the same for all *e*. We also evaluate **CASNet II** [45], an extension of CASNet with atrous spatial pyramid pooling [46].

#### 5.2.3. Weakly-Supervised Emotional Stimuli Detecter

As the baselines for the without-reference task so far are not designed for this task and are ignorant of emotion label *e*, we design a dedicated model, abbreviated as WESD (**W**eakly-supervised **E**motional **S**timuli **D**etection), using utterances in ArtEmis [11] for weakly-supervised training.

**Figure 9:**
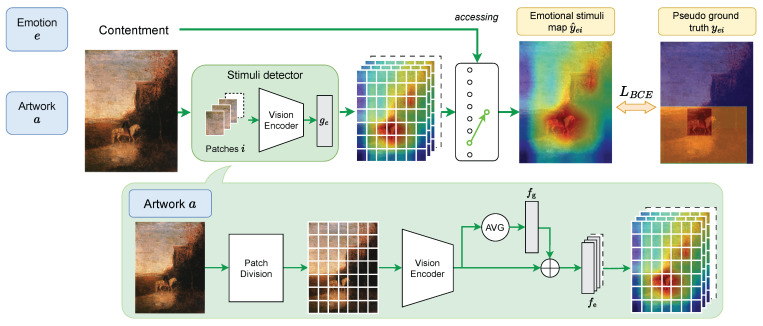
The overview of WESD. WESD predicts an emotional stimuli map for each emotion, and we access a certain map when the emotion (e.g., *contentment*) is given. For training, we use pseudo ground truth from CLIP + VinVL since APOLO does not have training data.

An overview of WESD is shown in Figure 9. It first uses a visual encoder, such as ResNet variants [47], that gives patch-wise visual features. The visual features of respective patches of artwork *a* are then fed into a binary classifier for each *e* to predict if the patch contains emotional stimuli for emotion *e*. Let $v_{i}$ be a feature vector for patch i∈K, where *K* is the total number of patches in one artwork. Classifier $g_{e}$ for emotion *e* predicts a score as

(6) $$\begin{array}{c} {\hat{y}}_{ei}=g_{e}\left(v_{i}\right)\in \left[0,1\right]. \end{array}$$

Specifically, $g_{e}$ predict s${\hat{y}}_{ei}$ by the feature of both the certain patch and the whole artwork, as

(7) $$\begin{array}{c} g_{e}\left(v_{i}\right)=F_{e}\left(v_{i}+F_{g}\left(\frac{1}{K}\sum_{k}v_{i}\right)\right), \end{array}$$

where $F_{g}\left(\cdot \right)$ is a fully-connected layer for embedding the whole artwork, and $F_{e}\left(\cdot \right)$ is a fully-connected layer to predict ${\hat{y}}_{ei}$. WESD contains multiple $F_{e}\left(\cdot \right)$’s and each of $F_{e}\left(\cdot \right)$ is related to a certain emotion *e* (e.g., *contentment*).

For training, ground-truth emotional stimulus *s* in APOLO can give direct supervision over $y_{ei}$; however, APOLO is only for validation and testing. We instead use a pseudo ground truth. We utilize CLIP + VinVL for the with-reference task to generate an emotional stimuli map $M_{e}$, which can be derived from the ArtEmis training set. This means that the predictions based on utterances are used to distill the knowledge about the emotional stimuli into $f_{e}$ for the without-reference (without-utterance) task. Emotion label *e* is only for identifying the map that has pseudo ground truth.

For this, we first divide $M_{e}$ into the same patches as $v_{i}$’s and compute the mean within each patch to obtain a soft label $y_{ei}$. The binary cross-entropy loss BCE(·,·) is used for training, i.e.,

(8) $$\begin{array}{c} L=\mathrm{BCE}({\hat{y}}_{ei},y_{ei}). \end{array}$$

For inference, WESD takes artwork *a* as the only input. The classifiers for all e∈E predict the score ${\hat{y}}_{ei}$, which is then summarized into ${\hat{y}}_{e}\in {\left[0,1\right]}^{B_{w}\times B_{h}}$, where $B_{w}$ and $B_{h}$ are the numbers of patches in the horizontal and vertical axes, respectively. The map ${\hat{y}}_{e}$ is then resized to the same size as *a* to obtain predicted emotional stimuli map ${\hat{Y}}_{e}$. Predicted segment $\hat{s}$ can be obtained by thresholding over ${\hat{Y}}_{e}$.

## 6. Experiments

*Metrics* For evaluation, we borrow the ideas from previous works to employ *bounding box* [30] and *segmentation* [31] scenarios, where the former only requires to roughly locate emotional stimuli, while the latter requires their precise shapes. The *bounding box* [30] evaluation focuses on both stimuli and their background (e.g., the emotion of awe in Figure 1), as it assumes that emotions are evoked not only by the stimuli but also by the background. While the *segmentation* [31] evaluation focuses on the stimuli (e.g., the human and the bears in Figure 1) themselves, as it assumes that the stimuli are more important than other regions to evoke the certain emotions. We use both methods, in considering that both could be related to the emotion elicitation process. For both scenarios, we calculate the precision with intersection over union (IoU) threshold θ (Pr@θ), as in [37,38,39,40,48,49]. We evaluate models with Pr@25 and Pr@50.

For baselines that output bounding boxes (i.e., FasterRCNN and VinVL), we collectively treat them as a single region (though they can be disconnected) for evaluation in the segmentation scenario. In contrast, for baselines that give segments by thresholding, we generate a bounding box for each connected component for the bounding box scenario.

*Implementation details* Our baselines in most cases use the default setting in the original paper. As for CLIP, we use the ResNet-50 variant throughout our experiments. For WESD, we resize artworks to 224×224 pixels. Bi-linear interpolation is used to resize ${\hat{y}}_{e}$ to ${\hat{Y}}_{e}$. We train the model for 20 epochs with batch size 128, learning rate $2\times 10^{-4}$, and decay 0.01. The model is optimized with AdamW [50]. For VilBERT [39] and 12-in-1 [40], we follow the procedure in the respective papers to fine-tune the models on refCOCO [37]. All training processes were performed on a Quadro RTX 8000 GPU, which took about 20 h for WESD.

All baselines require a suitable threshold to obtain segment prediction $\hat{s}$. We use the APOLO validation set to find the best one on it with IoU@50 and apply it for evaluation.

*Baseline variants* For the baselines with reference, which take utterances as input, we can instead use the emotion label (e.g., *excitement*), so that the models can find regions that can be associated with (or that the models learned to associate with) the word.

In addition to the baselines in Section 5, we evaluate the case where the entire artwork is predicted as $\hat{s}$.

### 6.1. Quantitative Analysis

The scores of all baselines for both with-reference and without-reference tasks are summarized in Table 3. We list our findings as follows.

**Artworks have something in common with natural images with respect to emotion.** For the without-reference task, VinVL, CASNet, and WESD work well. CASNet is the best among these three models in terms of Pr@25 in both bounding box and segmentation scenarios. It also hits the second-best in Pr@50 of segmentation. Although marginal, the superiority of CASNet may imply that EMOd [31] used for training the model in a fully supervised manner has something in common with APOLO. This is intriguing as regions in images that seem to be in very different domains (i.e., natural images and paintings in various artistic styles) share some characteristics. This insight may elicit further exploration of the connection between natural images and paintings, like studying the types of paintings for which a model learned from EMOd works.

**Emotional stimuli are highly correlated with objects and attributes.** The scores of region proposals by both FasterRCNN and VinVL are still comparable to CASNet and WESD. For the metrics that require precise localization (i.e., Pr@50) and segmentation, the gap seems slightly larger. We would say that emotional stimuli highly coincide with some objects. This is reasonable because the utterances (e.g., in Figure 1) mention some objects. A comparison between FasterRCNN and VinVL suggests the correlation between VinVL attributes [42] and emotion, which again makes a lot of sense.

**The domain of the utterances may be different from the text in typical vision-and-language tasks.** Interestingly, the scores of the with-reference task are mostly lower than those of the without-reference task. This is counterintuitive as the utterances should give beneficial information to identify the emotional stimuli. One possible rationalization is the domain gap. The utterances in ArtEmis [11,12] come with subjective statements (e.g., “*... bear looking forms leaves me uneasy.*” in the second utterance in Figure 1), which is not likely in typical vision-and-language tasks. This observation can be supported by the fact that the use of the emotion labels, which is very different from typical text, as input to the vision-and-language models worsens the performance (lines 7–9 versus lines 10–12 in Table 3). Additionally, the worst scores of 12-in-1 also can also support this because the 12-in-1 model is fine-tuned to 12 vision-and-language tasks and may lose the generalization capability for unseen tasks.

**WESD achieved better performance than CLIP + VinVL.** Regardless of the worse performance of CLIP + VinVL, WESD hit a higher performance than it, although WESD is trained from CLIP + VinVL. A possible explanation is that, despite the lower performance of CLIP + VinVL for individual artworks, there are some characteristics shared in the dataset, and WESD may capture them through training.

### 6.2. Qualitative Analysis

**Figure 10:**
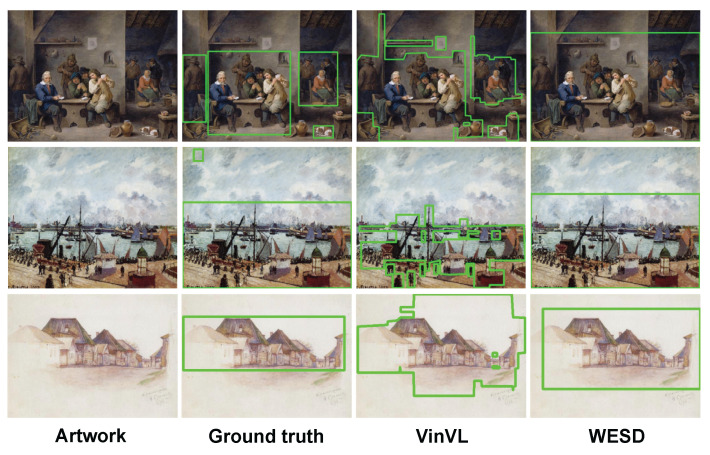
Examples of bounding box region detection. VinVL tends to distinguish objects exhaustively from the artwork, while WESD tends to find regions instead of certain objects.

**Figure 11:**
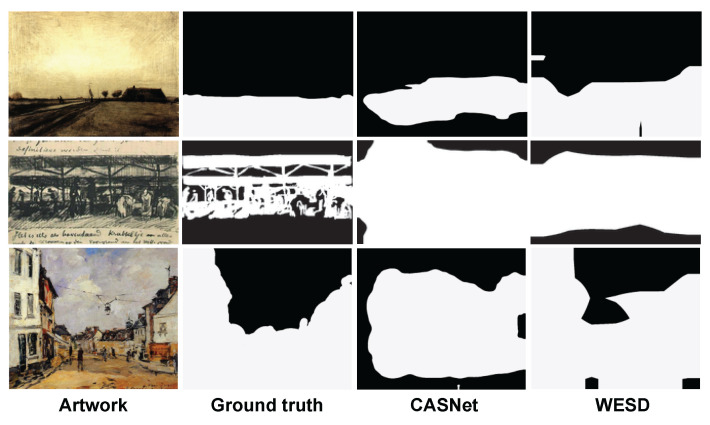
Examples of segmentation region detection. Compared to CASNet, WESD tends to find more related regions.

Qualitative examples are shown in Figure 10 and Figure 11 for the bounding box and segmentation scenarios, respectively. Figure 10 shows baselines with the top-two scores, i.e., VinVL and WESD, where VinVL’s bounding boxes are merged when they overlap. Since WESD makes bounding boxes that contain each connected segment of emotional stimuli, it tends to cover a large area. VinVL generates many small bounding boxes around objects, which coincide with the ground-truth bounding boxes.

Figure 11 compares WESD against CASNet. We find that CASNet tends to predict regions near the center of the image as emotional stimuli. This tendency is not surprising as the model is supervised by fixations from eye trackers and the image center seems to have a salient component. Meanwhile, WESD tends to find more relevant regions than CASNet, at least for these examples (although because the difference in the scores between WESD and CASNet is small, the trend is not consistent for the APOLO test set.

In general, detecting emotional stimuli in artworks is still challenging as none of the three models perfectly spot the emotional stimuli in both Figure 10 and Figure 11.

### 6.3. Emotion-Wise Analysis on Stimuli Detector

**Figure 12:**
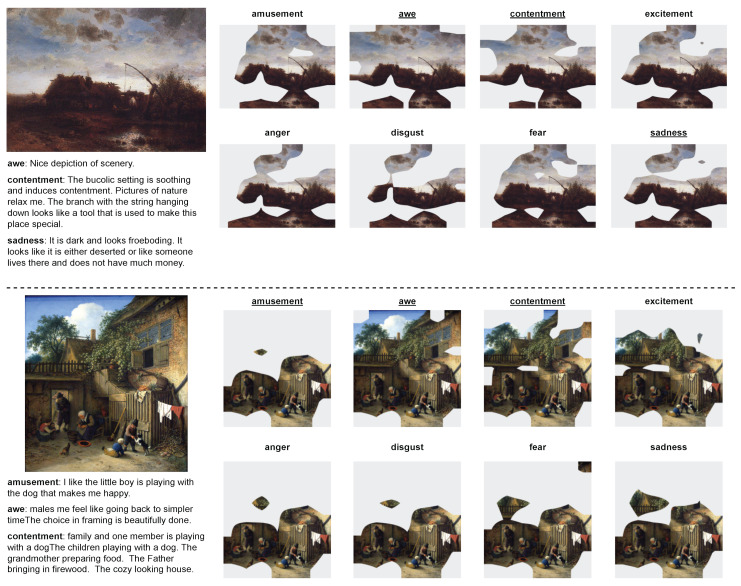
Examples of WESD’s prediction on eight different emotions. The texts on the left are the utterances from ArtEmis [11], which are not used during the prediction. The regions on the right are the predicted regions that evoke the corresponding emotions. The emotion tag on the right has an underline if this emotion appears on the left, i.e., has an annotation in ArtEmis.

In this section, we analyze how well WESD predicts emotional stimuli maps for each emotion in one artwork. Specifically, we use the artworks in the test set of ArtEmis for this experiment, and we ask WESD to predict the emotional stimuli map for all of the emotions. We only evaluate WESD as it is the only baseline model that is able to predict emotional stimuli maps for each emotion and does not need utterances as a reference. Some results are shown in Figure 12. Through our experiments, we have the following observations:

- **Predictions focus on similar regions.** Although the WESD’s predictions for each emotion are different, most of them focus on similar regions (e.g., the house and the pool in the first example and the people in the second example) in one artwork. The results could be reasonable as some regions in the artwork may play an essential role in evoking multiple emotions. We observe that such regions are also involved in the utterances.
- ***Awe* and *contentment* tend to involve more regions**. Compared with other emotions, *awe* and *contentment* usually involve more regions, such as the whole sky in the first example, and the building and tree in the second example. These results may be related to the factor that the emotions of *awe* and *contentment* are usually evoked by wider scenery in the artwork.

## 7. Emotional Stimuli and Deep Generative Models

We consider emotional stimuli detection a task that may benefit traing future deep generative models in generating emotional artworks, e.g., setting it as a loss function. In recent years, deep generative models, such as DALLE-2 [23] and Stable Diffusion [22], have demonstrated remarkable capabilities in producing high-quality images to users’ requirements. Such capacities also make these models popular in the artwork field, such as artwork generation [51] and editing [52].

In this section, we explore how much a popular deep generative model, Stable Diffusion [22], can handle emotions when generating artwork, and if our task and models can help improve its performance. To explore this, we randomly select 20 artists with one of their artworks in the APOLO dataset. Then, we make prompts by “The painting of *[artwork name]* by *[artist name]*, produce *[emotion]*”. We use Stable Diffusion v1.5 [22] to generate artworks for all combinations of 20 artworks and eight emotions, resulting in 160 generated artworks. Recently, DAAM [53] found that the aggregation of the cross-attention maps from Stable Diffusion can reveal the interpretation process of the model from prompts to images, i.e., reveal which parts of the image are related to a word in the prompt. We use DAAM to extract the internal attention map of *[emotion]*, which may indicate how Stable Diffusion interprets the emotion to the generated artwork.

**Figure 13:**
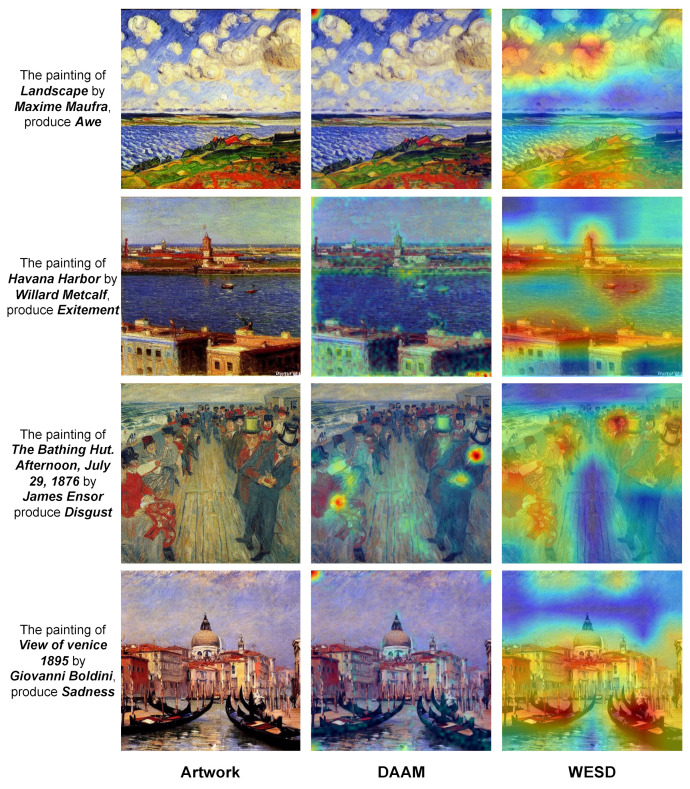
Examples of Stable Diffusion generated emotional artworks, the internal attention maps (DAAM), and WESD’s predictions. The texts on the left are the prompts for Stable Diffusion. WESD could be useful for Stable Diffusion to generate emotional artwork by guiding the model to emphasize the emotional stimuli.

We show some results in Figure 13. From the generated images, we find that Stable Diffusion can somehow generate artworks that can evoke certain emotions. However, from the internal attention map, we find that attention maps related to *[emotion]* are seldom focused. Instead, we observe that the attention sometimes focuses on the four corners of the artwork. These observations may indicate that it is still hard for Stable Diffusion to handle the relation between emotions and emotional stimuli. Compared to Stable Diffusion, WESD shows more concentration on the regions that are more related to the given emotions. The results may show a potential application of our work and WESD, to work as a guide and benefit Stable Diffusion in focusing on the emotional stimuli and generating more emotional artworks.

## 8. Limitations and Ethical Concerns

Our task is based on the appraisal theory of artworks and emotions [13,14]. Although this theory is reliable, it is continuously developing. We tried to remove inconsistent samples when constructing APOLO, as described in Section 4, but this may cause some domain gaps between our dataset and general artworks. Additionally, there are rising concerns about the ethical considerations of emotion recognition. As emotions are subjective and personal, trying to predict them with a machine learning model may be intrusive. We agree that emotion prediction could raise privacy issues and potential risks for model abuse. Being aware of this, we did our best to address these concerns proactively. In our experiments, we handled data responsibly and ensured that their use aligned with ethical standards. Additionally, we are planning to inform users of the inherent risks associated with our dataset and ensure they utilize it responsibly. Furthermore, we are prepared to take swift action, including freezing or deleting portions or the entirety of the dataset, if we identify any significant risks associated with its use. Through these measures, we hope to mitigate potential ethical risks and promote responsible usage of our research findings.

## 9. Conclusions

We introduced an emotional stimuli detection task that targets extracting regions from artworks that evoke emotions. For this task, we build a dedicated dataset, coined APOLO, with 6781 emotional stimuli in 4718 artworks for evaluation. We also provide APOLO with several baseline models to unveil the challenges in this task. Both qualitative and quantitative evaluations demonstrated that baseline models do not achieve a satisfactory performance, implying inherent difficulties in handling vague and abstract concepts of emotions. Furthermore, we explore how a deep generative model, Stable Diffusion, can handle emotions and emotional stimuli. We find that it is still hard for Stable Diffusion to understand and express emotions. We hope our work can bring inspiration to the fields of artwork analysis and visual emotion analysis. 

## Figures and Tables

**Table 1:** Summary of datasets for emotional stimuli detection. The source column indicates whether the images are from the social internet or artworks, while the ME (multi-emotion) column indicates whether an image has annotations for multiple emotions.

	Samples	Images	Source	Emotions	ME
EmotionROI [30]	1980	1980	social	6	No
EMOd [31]	1019	1019	social	2+1	No
APOLO	6781	4178	artwork	8	Yes

**Table 2:** Data structure of our evaluation set.

Painting	Emotion	Map_ID
a.y.-jackson_indian-home-1927	sadness	000000
aaron-siskind_new-york-24-1988	anger	000001
abdullah-suriosubroto_bamboo-forest	contentment	000002
abdullah-suriosubroto_mountain-view	excitement	000003
abraham-manievich_moscow-iii	excitement	000004
abraham-manievich_moscow-iii	sadness	000005
…	…	…

**Table 3:** Results of the evaluation on eight baseline models as well as a lower-bound baseline (i.e., the *Entire* artwork). From the left, *Task* tells that if a baseline model makes predictions with references (i.e., emotion tags or utterances). *Region proposal* shows if and which region proposal network is used by the certain baseline model. *Text input* shows the text input for generating emotional stimuli maps. *Multiple maps* is ✓ when the model can output multiple emotional stimuli maps for different emotions. For results in both *Bounding box* and *Segmentation*, we bold the best score and take an underline to the second-best result.

	Task	Baseline	Region Proposal	Input Text	Multiple Map	Bounding Box			Segmentation	
						Pr@25	Pr@50		Pr@25	Pr@50
1	w/o reference	Entire artwork	-	-	-	82.37	63.81		68.61	37.70
2		FasterRCNN	FasterRCNN	-	-	84.03	66.40		74.43	43.67
3		VinVL	VinVL	-	-	84.43	**67.54**		75.10	43.94
4		CASNet	-	-	-	**84.84**	66.02		**76.40**	44.15
5		CASNet II	-	-	-	**84.84**	63.59		76.24	40.18
6		WESD	-	-	✓	84.30	66.66		75.89	**44.97**
7	w/ reference	VilBERT	FasterRCNN	emotion	✓	82.17	63.08		72.14	39.64
8		12-in-1	FasterRCNN	emotion	✓	72.51	50.71		63.90	31.87
9		CLIP + VinVL	VinVL	emotion	✓	81.97	63.00		71.29	40.05
10		VilBERT	FasterRCNN	utterance	✓	84.10	65.41		75.26	42.18
11		12-in-1	FasterRCNN	utterance	✓	80.52	59.16		72.52	37.99
12		CLIP + VinVL	VinVL	utterance	✓	83.31	64.99		72.58	40.08

## Data Availability

Our data and methods are available at https://github.com/Tianwei3989/apolo.

## Abbreviations

The following abbreviations are used in this manuscript:

APOLO	**A**rtwork **P**rovoked em**O**tion Eva**L**uati**O**n
BCE(·)	Binary cross-entropy
IoU	Intersection over union
Pr@θ	Precision with IoU threshold θ
WESD	**W**eakly-supervised **E**motional **S**timuli **D**etection

## Nomenclature

The following nomenclatures are used in this manuscript:

*a*	Artwork
$B_{w}$	Number of patches in the horizontal axes
$B_{h}$	Number of patches in the vertical axes
$D_{t}$	Training set
$D_{v}$	Validation set
$D_{e}$	Test set
*e*	Emotion
E	Set of emotions
$f_{e}\left(\cdot \right)$	Emotional stimuli detector
$F_{g}\left(\cdot \right)$	Fully-connected layer for embedding the whole artwork
$F_{e}\left(\cdot \right)$	Fully-connected layer to predict emotional stimuli score ${\hat{y}}_{ei}$
$g_{e}$	Binary emotion classifier for emotion *e*
*i*	Patch
*K*	Number of patches in artwork *a*
$m_{r}$	Map of region *r*
$M_{u}$	Emotional stimuli maps for utterance *u*
$M_{e}$	Emotional stimuli maps for emotion *e*
*p*	Point/location of segment *s*
$P_{w}$	Points related to phrase *w*
*r*	Region of artwork *a*
*R*	Regions in artwork *a*, a set of region *r*
*s*	Segment of artwork *a*
$s_{w}$	Segment of phrase *w*
sim(·,·)	Similarity score
$\hat{s}$	Segment predicted by model
θ	Threshold of IoU
*u*	Utterance
$v_{i}$	Feature of patch *i*
*w*	Phrase
$W_{u}$	Phrases in utterance *u*
$y_{ei}$	Emotional stimuli score of patch *i* with emotion *e*
${\hat{y}}_{ei}$	Predicted emotional stimuli score of patch *i* with emotion *e*
